# Points of View and Readers’ Immersion in Translation: A Neurocognitive Interpretation of Poetic Translatability

**DOI:** 10.3389/fpsyg.2022.877150

**Published:** 2022-05-06

**Authors:** Qing Chen, Lin Shen, Shelley Ochs, Kairong Xiao

**Affiliations:** ^1^School of Interpreting and Translation Studies, Guangdong University of Foreign Studies, Guangzhou, China; ^2^Graduate School of Translation and Interpretation, Beijing Foreign Studies University, Beijing, China; ^3^Beijing Center for China Studies, Beijing, China; ^4^Beijing United Family Health Hospital, Beijing, China; ^5^College of International Studies, Southwest University, Chongqing, China

**Keywords:** poetry translation, cognitive neuroscience, cultural psychology, translatability, points of view, immersion

## Abstract

There have been few attempts at applying cultural neuroscience and psychology to the discussion of poetic translatability. This study employs cultural neuroscience and psychology methodologies and forms of evidence to explore the neurocognitive mechanisms by which cross-cultural variations in perspectives during the translation process influence poetic reception in the target culture. The English translation of Chinese poetry is often tasked with the supplement of perspectives and accompanied by cross-cultural variations of immersion. These changes have been substantially discussed from literary and poetic perspectives but remain understudied in terms of their neurocognitive and psychological implications. Through textual analysis of first-person points of view, this study attempts to apply neuroscience to the interpretation of the impact of differences in cross-cultural perspectives in poetry translation. Our findings suggest that a general tendency toward the supplement of first-person perspectives could boost the immersive experience by activating mirror neurons and the temporal parietal junction. These neuroscientific mechanisms underlying the observable cultural phenomenon offer implications for the translation of Chinese poetry in a way that generates brain responses and neurotransmitters similar to the source text. This study demonstrates how research in neuroscience can illuminate findings in cross-cultural communication.

## Introduction

The translatability, or untranslatability, of poetry is one of the most disputed issues in translation and linguistic studies. The working definition of translatability or untranslatability we are using in this paper is not a binary divide but is actually a spectrum, with untranslatability at zero and absolute translatability at 100. Scholars have adopted multiple theoretical approaches to analyze these issues (Large et al., 2019), including relevance theories (Xu and Gong, 2012), cross-cultural communication (Sun, 2012), factor analysis (Lee, 2012), otherness (Washbourne, 2015), dynamic equivalence (Lin and Zhao, 2017), and performability (Glynn and Hadley, 2021). However, a review of this literature shows that most studies include only macro-level analysis, with an emphasis on cross-culture differences.

Recently, with advances in cognitive neuroscience and their influence on multiple disciplines, academics have turned to focus more on micro-level analyses. The aim of these latter studies is to make invisible feelings “visible” through zooming in on brain reactions, since the understanding and reception of poetry and poetry translation can be traced to reactions in the human brain. On the other hand, the cross-culture work in academia allocates more attention to the topics of social behavior and attitudes in cross-cultural contexts, such as business models (Chin et al., 2021b,c), knowledge management (Chin et al., 2020, 2021a), and immigration psychology (Recupero et al., 2018; Taylor et al., 2021). Relatively few studies have been carried out on poetry translation, which demands that translators mitigate the cross-cultural differences, and this scarcity of research further impedes progress on this topic.

In view of the above, this study attempts to re-invigorate research on poetic translatability by introducing some findings in neurocognitive research that can be adopted as analytical lenses to be used in conjunction with literary narratology theories. Although these two are somewhat distant conceptually, one thing they share in common is an emphasis on point of view (POV). Narratology includes a detailed categorization of POV types that result from personal pronoun variations (Hühn, 2009, p. 115), while neurocognitive studies are more interested in investigating the mechanism by which POVs affect the sense of immersion, evidenced by both empirical evidence of related encephalic responses and qualitative reasoning (Gorisse et al., 2017; Medeiros et al., 2018; Monteiro et al., 2018).

Building on the findings from these two disciplines, and supported by in-depth textual analyses, we hypothesize that POV changes in translation contribute to the variation of immersion between source text (ST) readers and target text (TT) readers. When this difference is too great, this can be interpreted as poetic untranslatability. Such is often the case with the translation of Chinese classical poetry. When Chinese poems are supplemented with pronoun subjects in translation, which is a basic requirement of English grammar, then the sense of immersion is changed or deviates from the sense that the original produces. This can help explain why poetry is deemed untranslatable, regardless of how much the source and translated texts correspond in meaning or language, since the readers still experience a significant change in esthetic reception. In some cases, however, even if poems have undergone great changes in language during translation, even to the point of creating a new artistic creation, they can still produce a similar sense of immersion in target language readers. In this latter case, poetry is translatable despite great changes in meaning and language.

In this paper, we endeavor to explore whether variations in point of view may contribute to the untranslatability of poetry and whether this can be explained in neurocognitive terms. Specifically, we try to explore the following questions in the framework of neurocognitive theory on immersion. For topographical or landscape poetry, and for narrative poetry, respectively, what are the neurocognitive mechanisms related to immersion that can be correlated with the variation of POV? Which points of view induce untranslatability that be conceptualized in terms of immersion? And why, on the contrary, is translatability possible in poetry when the result is a hybrid or work of “transcreation”?

## Theoretical Framework: Personal Pronouns, Points of View, Immersion, and Their Correlation

Before we engage in further analysis, it is necessary to clarify the correlation between personal pronouns and points of view (POV), especially between first person pronouns and POV. The relationship between POV and the sense of immersion also needs to be clarified, and we should not assume a linear relationship between these two.

### Personal Pronouns and POV

Integrating views from narratology, cognitive neuroscience (Jacobs, 2015; Wittevrongel et al., 2018; Huang et al., 2019; Chen et al., 2020), and cognitive psychology (Arnheim, 1970; Chen et al., 2002; Carrasco, 2011, 2018; Wang et al., 2019), this study supports the notion that the perspective can be altered through word selection, a finding which has been verified by multidisciplinary studies. In the perspective theory of narratology, the determination of perspective is also based on, but not limited to, personal pronouns, that is, the same mode of visual focus, or point of view, may not always co-occur with the same personal pronouns (Prince, 1982). In particular, the first-person POV can be either an “internal” (diegetic) or “external” (extradiegetic) point of view (Fludernik, 2009). The former is an experiential point of view in which the experiencer narrates the event from an internal position and gains a perspective on the present. The external point of view is categorized into the retrospective perspective of the first-person protagonist (the narrator, as the protagonist, observes the past from his or her present POV), and the impersonal perspective of the first-person witness (also called camera-eye as the “I” in the narrative observes the event from a neutral perspective; Fludernik, 2009). Different personal pronouns change the POV and are associated with different modes of focus (More specific relations between POV, personal pronoun, and vantage point can be seen in Table 1).

**Table 1:** The relationship between person pronoun and point of view.

Point of view (Perspective)	Person pronoun	Vantage point
Single-Layered POV of the First-Person Experiencer	First-person pronouns (Present tense)	The experiencer’s diegetic present POV
Multiple-Layered POV of the First-Person Reflector	First-person pronouns (Past tense)	The experiencer’s diegetic past POV + present POV
Multiple-Layered POV of the Second- and Third- Person Narrator	Second- and third- person pronouns	The narrator’s extradiegetic POV + The experiencer’s diegetic POV

Source: Compiled by the authors with the previous findings on narratology (Prince, 1982; Fludernik, 2009). The figure above displays the relations between personal pronoun, vantage points, and POV and lays the foundation for the analysis in the following sections. The links between personal pronouns and POV provide the premise for the hypothesis that different person pronouns reflect different modes of POV and thus generate different levels of immersion.

Studies in cognitive neuroscience and psychology also confirm that pronoun selection impacts the construction of POV. The brain responds to textual features of poetry translation and forms an integrated perception based on previous experiences, which then enables the interpretation of meaning. First-person pronouns may indicate either a single perspective or a nested multiple-layered perspective. For the former, the first-person subject is the experiencer. As for the latter, the first-person subject is the past “self” of the experiencer instead of the current “self.” With second-person or third-person pronouns, the subject of perspective is not the experiencer, thus creating a multiple-layered perspective that entails both the narrator’s extradiegetic POV and the experiencer’s diegetic POV. In the multiple-layered perspective or the zero focalization (Hühn, 2009, p. 115), the perspective of the subject can also be collapsed into the experiencer’s perspective because it resonates or is consistent with it.

Combining the theories on POV from neurocognitive studies, visual psychology, and narratology, the relationship between personal pronouns and POV can be summarized into four types as follows.

### POV and Immersion

Neuroscientific, empirical analyses generally concur that the first-person internal POV can generate a stronger sense of immersion than the first-person external POV or the second- or third-person POV (Salamin et al., 2010; Debarba et al., 2015; Denisova and Cairns, 2015; Gorisse et al., 2017; Medeiros et al., 2018; Monteiro et al., 2018). More specifically, the first-person POV generates a sense of imagination that draws the reader closer to the experiencer while the third-person perspectives induce more calm and objective thoughts. The reason, according to psychology, lies in the role of first-person POV in generating immersive imagination, as it is associated with identification, which is an unconscious process of imagination in response to external pressure (Freud, 2003) and constitutes an important part of “superego” in Freudian terms. This process has significant implications for this study, because we know that in cross-cultural contexts’ identification is defined as an imaginative process that is triggered by the fictional character (Cohen, 2001, p. 250) that helps form immersive experiences (Jacobs and Lüdtke, 2017) that can transcend one’s native culture. Therefore, the evidence from psychological theories confirms that POV is connected with the sense of immersion.

The connection is also supported by neuroscientific studies that employ different approaches yet reach similar conclusions. If we examine this process of identification from the perspective of neuroscience, we can see that underlying neuroscientific mechanisms are intimately related to mirror neurons, which are found in the F5 section of the premotor cortex (Rizzolatti et al., 1996). This finding has been confirmed by empirical neuroscientific studies that test the correlations between imagination and behavior (Parsons, 1994). In other words, similar neurons are activated when we imagine certain behavior compared to when we actually engage in such behavior. The finding has also provided a biological basis for embodied cognition studies (Shapiro, 2019, p. 105), which hold that “mental functions” in general depend upon the “material and temporal details of their implementation” (Cappuccio, 2019, p. 18).

In addition, studies on digital gaming also confirm the relationship between the player’s point of view or perspective and his or her sense of immersion (Kilteni et al., 2012; Cairns et al., 2014). These studies compare the immersion generated by first-person versus third-person perspectives in gaming. Neuroscientific evidence suggests that the first-person point of view (or the “experiencer” perspective) can promote the integration of the player’s real and virtual “self” (Salamin et al., 2010; Debarba et al., 2015; Denisova and Cairns, 2015; Gorisse et al., 2017; Medeiros et al., 2018; Monteiro et al., 2018). In conclusion, neuroscientific research on both identifications when reading literature and engaging in digital gaming has shown that first-person perspectives can trigger more in-depth immersion. In other words, the first-person POV, compared with second- or third-person perspectives, produces a stronger sense of immersion.

## Data for Analysis: Determination of POV in Translation of Classical Chinese Poetry

Chinese is a language in which the subject can often be omitted. Classical Chinese poetry shows an even stronger tendency to omit the subject, and this is, in fact, one of its key features that stands in contrast to modern, written Chinese (Cai, 2008). The lack of subject in many ancient poems means a lack of a defined point of view, giving the readers freedom to adopt the first-, second-, and third-person perspectives. This poses major challenges for English translators, as English is a subject-prominent language. In the translation of classical Chinese poetry into English, adding a subject is often necessary. However, translators have a certain degree of freedom and initiative in the selection of perspectives or points of view. The different personal pronouns they choose will express different modes of focus. Among the choices for pronouns, the first-person seems to be the favorite of both Chinese and overseas translators over the decades, something which is quite evident through statistical analysis of the collection this study uses as our database of cases.

This study primarily analyzes a collection of English translations of Chinese classical poems, *A Comparative Study on English Translations of Old Gems,* which has been frequently cited in Chinese academia. It was edited by the renowned Chinese linguist and translator Lü Shuxiang in 1940s, and in 1980s, he re-edited the collection with Xu Yuanchong, another very distinguished poetry translator. The poems selected are highly representative of classical Chinese poetry and cover a wide time span; the translators are from multiple countries, and most of the translated works have achieved far-reaching influence. While we limit our discussions in this paper to Lü’s collection, our conclusions were informed by consideration of many other works. Here is a preliminary statistical overview of the collection in terms of person pronouns. Of the selected 100 poems, 50 poems omit the subject in the original Chinese version. Of the 227 translations of these 50 ancient poems, about 150, or 66%, adopt the first-person perspective. Among the translations with first-person perspectives, the ones with the experiencer’s present point of view occupy the majority at more than 50%. The rest of the first-person points of view in the Chinese-English translations are from first-person multiple-layered, second-person, and third-person perspectives.

Generally speaking, academics believe that this kind of supplementation of subject pronouns causes cultural, esthetic, and emotional losses (or at least alterations) in the translation of poetry, leading many people to conclude that poetry is untranslatable or lost in translation (Catford, 1965, p. 35; Qian, 1981, p. 18; Liu, 1985, p. 31; Jakobson, 1987, p. 429; Bassnett and Lefevere, 1998, p. 58; Wang, 2001). Some scholars even hold the belief that poetry translation requires so many changes that it betrays the original to the point that it is, in fact, a new creative work and no longer a translation.

More specifically, in Chinese-English translation, the discussions of researchers and translators have been centered around translation strategies or cross-cultural esthetic losses. A great number of researchers opine that the determination of POV by supplement of person pronouns “clarifies the previously ambiguous relationships and causes the shrinkage of space for imagination and re-creation” (Huang, 2006, p. 154). Generally, academics consider the supplementation of subjects to be the primary reason behind the cultural, esthetic, and emotional losses, and, ultimately, untranslatability. The notion of untranslatability is held by many (Catford, 1965, p. 35; Qian, 1981, p. 18; Liu, 1985, p. 31; Jakobson, 1987, p. 429; Bassnett and Lefevere, 1998, p. 58; Wang, 2001), who claim that translated poetry is dead and has lost its vitality. In other words, they regard the variations in poetry translation a betrayal of the original poem and argue against the transcreation of poetry.

Yet interestingly, many translations, though literally quite distinct from the original, and therefore considered to be a form of “creative treason,” are nonetheless able to convey the image, essence, or spirit of the poem in question (Escarpit, 1971; Xie, 1999; Sun, 2001). For example, the Cathay (华夏集) by Ezra Pound, a transcreation of classical Chinese poetry, is applauded by Eliot (1954, p. 14) who praise Pound as the “inventor of Chinese poetry for our time” who allows readers to “really at last get the original” (Tsang, 2014; Xie, 2014; Jatobá, 2019). There is a consensus that literal renderings always fail to create equivalent responses in the readers of the target text. This phenomenon is often interpreted from cultural perspectives and in macro speculations. The micro-level analysis of the mechanism behind the lack of equivalence by which how culture influences the generation of responses has yet to be delineated. This paper, therefore, is intended to present more in-depth interpretations at a micro-level through detailed textual case analyses and speculation about the neurocognitive responses of readers to translated poetry.

## Case Analysis: Neurocognitive Interpretation of Untranslatability and Translatability

Based on a detailed analysis of the POV changes in poetry translation, the following textual analysis focuses on the untranslatability of topographical poetry and narrative poetry, and the possibility of translatability through poetry “transcreation.”

### Untranslatability of Topographical Poetry: Cross-Cultural Variations of Immersion

Classical Chinese poetry, especially poetry from the Zen Buddhist tradition, often conveys feelings through the depiction of scenery. The point of view is often vague when read by a native speaker. The point of view is indeterminate when the reader reads the poem, and the focus of the reader is inconstant throughout the cognitive process, shifting between internal and external POV. This feature of indeterminate focus is shared by classical Chinese paintings, which adhere to the principle of using “three vantage points” in one drawing. Faced with the shifting visual focus, translators often supplement with a first-person subject, defining the POV as a first-person one. The use of the first-person POV can bring about more integrated imagination, triggering a deeper fusion of the real self and the virtual self, and giving readers a more immersive esthetic experience. In terms of cultural psychology, it enhances the sense of immersion while limiting the dissociative state of the original perspective. This relates directly to the unavoidable untranslatability in question here. A shifting focus, or the absence of a clear focus altogether, is especially common in the Zen Buddhist poetry that attaches great emphasis to the integration of human beings into natural environments.

The following poem is representative of poems on natural scenery and the principles of Zen Buddhism from the Tang dynasty (618–907 CE), China’s golden age of poetry. The ambiguity of the perspective, the hidden subject, and the uncertainty of the relationship between the observer and the observed object reflect the spirit of the poem, which is “to forget oneself while appreciating the mountains and rivers” (Zhou and Liu, 2004, p. 161). The absence of pronouns implies an indefinite focus. In particular, the first sentence indicates only the time and action, leaving the subject of the action to be specified. Who is the observer of this early morning scene? This question is left for readers to answer. Therefore, the readers may have many different answers based on their own understanding and feelings. The first four sentences of the original poem can be interpreted as either an external perspective of an observer outside the scene or an internal first-person perspective of the experiencer inside the scene. Although the last four sentences clearly reveal the observer’s internal thoughts, it is still uncertain whether the observer is an omniscient narrator or an experiencer in the scene. But, interestingly, four of the five translations add the first-person experiencer perspectives. Here are the five translations of the sentences to be discussed as:

[Poem A]: 清晨入古寺，初日照高林。曲径通幽处，禅房花木深 (常建《题破山寺后禅院》).

[Pinyin] qīng chén rù gǔ sì, chū rì zhào gāo lín. qǔ jìng tōng yōu chù, chán fáng huā mù shēn (tí pò shān sì hòu chán yuan by cháng jiàn).

[Literral Translation] Early in the morning into the ancient temple, early sunshine enters the tall forest. The winding path leads to the secluded place, and the Buddhist temple has deep flowers and trees.

(A1) At dawn I come to the convent old, While the rising sun tips its tall trees with gold. As, darkly, by a winding path I reach Dhyana’s Hall, hidden midst fir and beech

(Translated by Herbert A Giles; Lü and Xu, 1988, p. 221).

(A2) I come to the old temple at first light; Only tree-tops are steeped in sunbeams bright. A winding footpath leads to deep retreat; The abbot’s cell is hid’ mid flowers sweet

(Translated by Xu Yuanchong; Xu Yuanchong, 2012, p. 64).

(A3) In the pure morning, near the old temple, Where early sunlight points the tree-tops, My path has wound, through a sheltered hollow of boughs and flowers, to a Buddhist retreat

(Translated by Witter Bynner; Lü and Xu, 1988, p. 222).

(A4) At first light I entered the ancient temple As the high woods shone in the sunrise. Winding ways had led me to this quiet place. An Abbot’s Cell deep in trees and flowers

(Translated by Innes Herdan; *Ibid*: 222).

(A5) Where the sun’s eye first Peers above the pines, On the ancient temple, early daylight shines. To retirement guiding leads the winding way Round the Cell of Silence Flowers and Foliage stray

(Translated by W. J. B Fletcher; *Ibid*: 221).

The first four of the five examples above all adopt the first-person POV. As noted above, the same personal pronouns do not necessarily indicate the same perspective. The four translated poems can be divided into two different categories from the perspective of cultural neuroscience based on the different tenses employed here. The first three translated versions adopt the simple present tense and focus on the experience of the event with the use of the first-person experiencer POV. In the translated version (A4), the past tense is used in the translation of the first four sentences, but the last four sentences are in the present tense. It implies that the narrator “I” is recalling the past, and since the present “I” is already out of the past, we can conclude that the last four sentences are using the multiple-layered reflector POV. The last four sentences are translated into the present tense, which brings the reader back to the past, and the translation of these four sentences is transformed into the experiencer’s experience. In other words, the translation of the poem displays a switch between the single-layered and multiple-layered perspectives. Unlike the first four translated versions, there are no personal pronouns in the translated version (A5). Like a camera, the observer objectively “scans” the scene outside the Zen room. The omission of “entrance” in the translation also indicates an objective multiple-layered POV.

The five examples are categorized into three types. The first three versions fall into type 1 that feature the single-layered experiencer POV. The translated version (A4) belongs to type 2 characterized by a shift between single- and multiple- layered POV. The translated version (A5) is in type 3, which features third-person POV. In the translated versions (A1) and (A2) of type 1, the first line of the translation clearly places the first-person narrator “I” in the picture, and all the scenery is the object of “I.” This POV forces the reader to observe the scenery in the eyes of “I” and through the experience of “I” to feel the changes of time and space. In contrast, as shown in the Table 2, the first-person pronouns in the translated version (A4) of type 2 appear most frequently, and the four pronouns strengthen the personal experience of “I.” It is also the version with most obvious shifts within the same first-person perspective. These shifts from the narrator’s present feelings to his or her review of the experience create a feeling of in-depth immersion. More specifically, as the poem starts with “my” entry and ends with “my” auditory judgment, “winding ways” is an extension for “I,” and the “pool” is used to “clear my minds,” which enhances the sense of immersion. At the same time, the shifts of POV will also bring about cognitive transformation and even changes in self cognition, which has been demonstrated by a number of cognitive neuroscientific experiments (Vogeley et al., 2001, 2004; Vogeley and Fink, 2003; Seger et al., 2004; David et al., 2006; Northoff et al., 2006). As for type 3, example (A5) is obviously cautious in this regard. Throughout the version (A5), no personal pronoun exists, completely excluding the characters from the picture. The scenery, as the “protagonist” of the poem, is directly presented in the poetry and dominates the whole poem, thus creating a sense of distance between the reader and the narrator in the poem.

**Table 2:** Comparison of POV between five translated versions of the Zen Buddhist poem.

Translation	Person pronoun	Tense	Point of view (Perspective)	Vantage point
A1	I, I	Present tense	Single-layered POV of the first-person experiencer	Internal (diegetic) perspective
A2	I, I	Present tense	Single-layered POV of the first-person experiencer	Internal (diegetic) perspective
A3	My	Present tense	Single-layered POV of the first-person experiencer	Internal (diegetic) perspective
A4	I, me, our, I	Past tense to Present tense	Multiple-layered POV of the first-person reflector to single-layered POV of the first-person experiencer	External (extradiegetic) perspective to internal (diegetic) perspective
A5	None	Present tense	Multiple-layered POV of the third-person narrator	External (extradiegetic) perspective

We may see from the discussions above that when the subject is supplemented in translation, the original vagueness of POV becomes defined, and the uncertainty of focus becomes determined. However, Chinese poetry focuses on natural scenes because this is a way of expressing something about human beings without making them the literal subject of the poem; the beauty lies in the ambiguity. Once the point of view is determined, the sense of immersion will change, and this is part of the reason why theorists deem poems untranslatable. On the one hand, the first-person point of view, especially the first-person experiencer point of view, boosts immersive experience. At the same time, the readers’ sense of participation is enhanced. “Subjects and objects are in agreement with each other in the ontological sense” (Zhou and Liu, 2004, p. 161), which accentuates the existence of the subject of “I.” To a certain extent, it actually changes the hidden self and the ego in terms of cultural psychology, which also contributes to the untranslatability of poetry in cross-cultural communication.

On the other hand, the intentional choice of third-person pronouns over first-person ones might weaken the sense of immersion in reading and make the readers feel as if they were watching a painting without being physically present, which aggravates the antagonism between humans and nature. Culturally speaking, the translated poems are further away from the Chinese concept of the unity of humans and nature. In a word, the differences in POV between the original poem and the translated version might either limit or broaden readers’ cultural perception, triggering different sense of immersion, which constitutes an important factor in untranslatability.

### Untranslatability of Narrative Poetry: Cross-Cultural Variations of TPJ Activation and Character-Based Immersion

In addition to immersion, cognitive neuroscience studies on narratives have also found links to the temporo-parietal junction (TPJ) in the brain. One study suggests that the TPJ plays an important role in inferring the protagonist’s thoughts (Yang, 2020, p. 160–162). The most compelling evidence supporting the hypothesis comes from looking at subjects’ ability to perform reasoning about other people. In this major piece of research, short story experiments are conducted to test people’s responses to stimuli. They found that the TPJ is an important cortical structure for story comprehension which enables the listener to step into the shoes of the protagonist in the story. In one experiment described by Saxe and Kanwisher (2003), participants read short stories of five types:

a. false belief stories in which the subject’s belief conflicts with the real world;
b. false photograph stories in which the photograph does not match the real world;
c. desire stories in which the story describes the individual’s desire;
d. non-human description stories in which non-animal objects are simply described; and
e. physical people stories in which the physical characteristics of the characters are described.

It was found that the activation intensity of bilateral TPJ was about the same in false-photograph stories as it was in false-belief stories. In addition, with the stimuli of desire stories, the activation intensity of the left TPJ was higher than the baseline level and lower than that of the false belief story. Importantly, the bilateral TPJ showed negative activation under the stimuli of non-human description stories and physical human stories. These findings reveal the importance of TPJ to readers and listeners in inferring the mental state of the protagonist.

Based on this research, we can see that the closer a story is to a person’s mental state, the more likely it is to activate TPJ. Since the difference of the personal perspective in the narrative is related to the differences in the sense of immersion, it can be hypothesized that the difference of POV is a factor in subjective psychological intensity. Therefore, the same poem, translated from different perspectives in terms of time and persons, might generate different levels of TPJ activation intensity. Furthermore, it can be inferred that the original perspective of poetry is not fixed and therefore, it is possible to make creative choices in translation. Cultural and language differences force the translator to narrow down the choice to a specific perspective. This shift from indefinite focus to a determined POV, from the perspective of neuroscience, may generate a difference in the activation of TPJ between the target readers and the original readers, which may explain translatability or untranslatability.

The following analysis is of another poem from the Tang dynasty by poet Wang Jian, describing a bride’s emotional life after she gets married and moves into her husband’s house. In ancient China, a woman lived in her husband’s house after marriage and had to do housework and take care of her mother-in-law beginning 3 days after her marriage. This poem describes the mental state of a bride at this time. Though some academics conjecture that the poem is in the third-person perspective, the original poem probably has no subject and therefore an uncertain perspective. However, most of the English translations adopt the first-person point of view, with only a few opting for the third-person perspectives (Huang, 2006, p. 153). The three representative translated versions recommended by Huang Guowen, a contemporary theorist of translation studies, are as follows:

[Poem B] 三日入厨下，洗手作羹汤。未谙姑食性，先遣小姑尝 (王建《新嫁娘词》).

[Pinyin] sān rì rù chú xià, xǐ shǒu zuò gēng tang. Wèi ān gū shí xìng. xiān qiǎn xiǎo gū cháng (xīn jià niáng cí by wáng jiàn)

(B1) Married three days, I go shy-faced. To cook a soup with hands still fair. To meet my mother-in-law’s taste, I send to her daughter the first share (A Bride)

(Translated by Xu Yuanchong; Huang, 2006: 153).

(B2) The third day I went into the kitchen, Washed my hands and made the soup. Now yet sure of my mother-in-law’s tastes, I sent some first for sister-in-law to try (Words of the Newly-Wed Wife)

(Translated by Burton Watson; Ibid: 153).

(B3) She comes to kitchen, married but three days, Having washed hands, she makes some consommés. Knowing her mother-in-law’s taste not yet, She let young sis have first, a clue to get (A Newly Wedded Daughter-In-Law)

(Translated by Wang Dalian; Ibid: 153).

In terms of narratology, translated version (B1) uses the first-person experiencer POV from an internal vantage point, and version (B2) is the first-person experiencer’s retrospection of the past from an external vantage point. Version (B3) uses the third-person omniscient POV—i.e., a typical external perspective. The external perspective suggests that the narrator is observing from the outside, while the internal perspective suggests that the narrator retrospects from the inside. The former tends to show calm feelings, while the latter tends to display more subjective emotions (Shen, 2009, p. 100). Therefore, translation (B1), which adopts the first-person internal perspective, displays the most personal subjective emotional overtones among the three versions and the strongest mergence between the narrator and the experiencer. In this translation, it is easiest for the reader to directly experience the strong impact of the “self” through the eyes of the experiencer.

Corresponding to the five story types in the experiment of Saxe & Kanwisher, the translated version (B1) falls into the type (c) of the desire story, while translation (B3) is close to the (e) physical human story type. Translation (B2) is in between the two. We can put forward a bold speculation that among the three translated poems, the readers of version (B1) are more likely to have the highest activation level of TPJ, while version (B3) tends to generate the lowest level. As seen from the comparison above, the difference of POV in different translated versions may play a role in generating the different levels of activation of TPJ. The difference might explain untranslatability to some extent. In other words, the choice of the POV will not only influence the sense of immersion but also the activation level of TPJ, and it can thus be hypothesized that POV is an important driving force forming the untranslatability of narrative poetry.

### The Interpretation of Translatability: “Wrong” Words but the “Right” Neurons

Regardless of whether we use the methodology of cultural psychology or other theories on immersion, the general principles of translatability or untranslatability are quite similar. First, in terms of cultural psychology, the impact of culture on cognition has long been the consensus of neuroscience and has been confirmed by multiple empirical studies. The construction of POV is influenced by cultural experience (Crick, 1994), and social and reading experience is needed to form a complete schema. For example, due to the role of culture in shaping the neural connections of the brain, when the same work is read by source language and target language readers, the difference in esthetic experiences might induce different levels of activation of visual neurons. For example, if the color “red” appears in *A Dream of Red Mansions* with obvious Chinese cultural connotations that are different in the target language culture, translators usually should and usually do adopt the strategy of free translation to avoid causing erroneous imaginations.

Second, in terms of immersion, since cultural experience restricts the construction of perspective, the sense of immersion will also be restricted by culture. According to the theory of self-referential processing (Gazzaniga et al., 2011), readers who grow up in different cultures display weaker self-referential effects related to unfamiliar cultural factors which are less relevant to themselves, and these cultural influences are connected with a shallow level of information processing to some extent.

The analysis from these two perspectives may shed light on a new understanding of transcreation and the related issue of translatability. Many academics think that “transcreation” means the “new” work has a certain level of distinctness from the original work and is a re-creation rather than a translation, such as Pound’s *Cathay*. However, from the perspective of cultural neuroscience, although the translated poetry has undergone great changes at the language level, it is still possible to activate areas of the brain in similar ways as the original poetry, thereby stimulating similar emotions and achieving cognitive equivalence. For example, in “Ode to the West Wind,” the “west wind” is changed to “east wind” in some translations because “west wind” in British culture has connotations of warm and pleasant feelings as “east wind” does in Chinese culture, and theoretically activates brain regions associated with positive emotions and produces neurotransmitters related to warmth. Although “east wind” deviates from the original poem at the language level, it can adapt to the target language culture, activate the same brain regions in the target language readers, produce similar neurotransmitters, and bring the similar feelings of warmth and joy. In this sense, poetry is translatable because the “wrong” words in translation activate the “right” neurons in target readers.

Of course, this kind of translatability does not always involve language-level changes. Poetry translation with words that are close to the original, literal text might also enable the original and target readers to activate similar brain areas and to enjoy similar esthetic experiences or sense of immersion. The condition is that the readers of the original poem and the readers of the translated poem have common situational and cultural experiences, making it possible to activate the common brain area under similar descriptions. This especially applies to poems depicting nature or other phenomena that generate few culture-specific understandings. For example, a sentence in Wang Wei’s *Envoy to the Frontier* presents a scene of the desert’s spectacular sunset. The lines go, “In boundless desert lonely smokes rise straight; over endless river the sun sinks round”^1^ (Translation by Xu Yuanchong). Just as Chinese readers are stunned by horizontal and vertical contrast and tension, the English readers most possibly feel the same for this piece of poetry. As the natural environment is the common visual and environmental experience of almost all human beings, it is highly possible that similar linguistic descriptions can activate the same brain regions. Of course, the word “same” here only means a high degree of coincidence and not absolute sameness. Nevertheless, individual experience varies from person to person. As the saying goes, there are “a thousand Hamlets in the eyes of a thousand readers.”

To sum up, as we view cross-cultural communication and cultural contexts through the lens of cognitive neuroscience, we find that the use of inequivalent language may help render poetry in a way that achieves equivalence and translatability. Simply put, the neuroscientific mechanism behind this lies in the activation of similar brain sections. In this sense, the “transcreation” considered by many a treason to the source text is actually conveying equivalent emotions in a neuroscientific sense. With “wrong” words showing major differences from the source text, the translation is, however, able to activate the “right” brain sections, neurotransmitters, and neurons. The readers of the target text have the same neurological reactions to the translation as the readers of the source text. The literally “wrong” translation might be a correct interpretation of the poetry at hand and can achieve successful cross-cultural communication from the perspective of neuroscience.

## Conclusion

Poetry is considered by many to be the most concise literary form for conveying human emotions. The choice of the first-person perspective in the English translation of Chinese poetry is not only in accordance with the linguistic norms of the target language but is also a reasonable strategy for expressing poetic emotion and generating an immersive experience. Language and culture constitute two-way communication. Language reflects and is subject to culture, while language creates, shapes, and influences culture. As a cross-cultural communicative activity, translation also shows its constructive power in cross-cultural contexts. When classical Chinese poetry is translated into English and other languages, the poetry is taken out of the original cultural context in terms of both time and space. It is difficult for most readers of the translated poem to experience and understand poetry in the context of the source culture as the readers of the source language do. They can only construct their own cultural context through the translation to build esthetic experience by perceiving or speculating on the source culture. This process has previously mainly been studied on the macro-level, with concepts pertaining to that level of analysis, but it is now possible to borrow neuroscientific findings and methods to carry out micro-level investigations, as exemplified in our study.

As shown in our case analysis, through neuroscientific or neurocognitive findings and methodologies, cross-cultural poetic translatability and untranslatability may be fruitfully understood and explained. On the one hand, the supplementation of subjects and variations of perspectives constitute part of the phenomena of untranslatability. From the perspective of neuroscience, untranslatability can be fruitfully explained by the neuro mechanisms of immersion, mirror neurons, and TPJ responses. Borrowing from the methods of neuroscience enables a more in-depth, micro-analysis that has been only minimally explored in the existing literature. On the other hand, translatability is neurocognitively possible, especially when considered in terms of cultural psychology and immersion.

Although the hypotheses proposed in this study are limited by a lack of experimental studies, this study puts forward the intriguing proposition that neuroscience can contribute significantly to poetry translatability analysis, laying a foundation for the interdisciplinary study of cultural neuroscience and poetry translation. More empirical studies on the reception of poetry translation from the perspective of neuroscience will enrich our understanding of psychological reception. The possibility that cross-cultural communication may be improved by in-depth neuroscientific analysis renders its exploration worthwhile. We hope that the neuroscientific hypotheses on the causes of, and solutions to, “untranslatability” put forward by this study will encourage other researchers to explore this type of interdisciplinary study.

## Author Contributions

QC designed the research and wrote the main part of the manuscript. LS and SO offered modification suggestions and helped translating. KX provided guidance throughout the entire research process. All authors contributed to the article and approved the submitted version.

## Funding

This research has been supported by the 2019 Project “Emotion and Affection in Poetry Translation: Cognitive Analysis upon Affective Computing” of Guangdong Planning Office of Philosophy and Social Science (Project No. GD19CYY09) and the 2019 Project “Entrepreneurship education for cyber-literature translators” of Guangzhou Municipal Education Bureau (Project No. 2019KC212).

## Conflict of Interest

The authors declare that the research was conducted in the absence of any commercial or financial relationships that could be construed as a potential conflict of interest.

## Publisher’s Note

All claims expressed in this article are solely those of the authors and do not necessarily represent those of their affiliated organizations, or those of the publisher, the editors and the reviewers. Any product that may be evaluated in this article, or claim that may be made by its manufacturer, is not guaranteed or endorsed by the publisher.

## Footnotes

^1^Source text: 大漠孤烟直，长河落日圆 (王维《使至塞上》).
